# Uncommon Territory: Multifocal Tumor-like Brain Lesions in Granulomatosis with Polyangiitis

**DOI:** 10.3390/diagnostics16142277

**Published:** 2026-07-21

**Authors:** Ana Petkovic, Ana Drazic, Marija Jovanovic, Snezana Arandjelovic, Aleksandra Plavsic, Rada Miskovic

**Affiliations:** 1 Center for Radiology, University Clinical Centre of Serbia, 11000 Belgrade, Serbia; ancipetkovic@gmail.com (A.P.); macvanskimarija@yahoo.com (M.J.); 2 Faculty of Medicine, University of Belgrade, 11000 Belgrade, Serbia; snezana.arandjelovic2019@gmail.com (S.A.);; 3 Clinic of Allergy and Immunology, University Clinical Centre of Serbia, 11000 Belgrade, Serbia; drazic998@gmail.com

**Keywords:** granulomatosis with polyangiitis, central nervous system, intracerebral granuloma, tumor-like brain lesions

## Abstract

Granulomatosis with polyangiitis (GPA) is a necrotizing granulomatous vasculitis affecting predominantly small vessels, typically associated with PR3-ANCA positivity. Central nervous system involvement beyond cranial nerve palsies is rare, with intracerebral granulomas being exceptionally rare and lacking specific imaging characteristics, often mimicking malignancies or other intracranial pathologies. This significantly complicates the diagnostic process, particularly in the absence of active systemic disease. We report a case of a 71-year-old woman in whom neurological symptoms and magnetic resonance imaging revealed intracerebral granulomas and led to the diagnosis of GPA. Early recognition of tumor-like brain lesions in GPA and prompt initiation of immunosuppressive and anti-edematous therapy are crucial to prevent progression and potentially life-threatening compressive complications.

**Figure 1:**
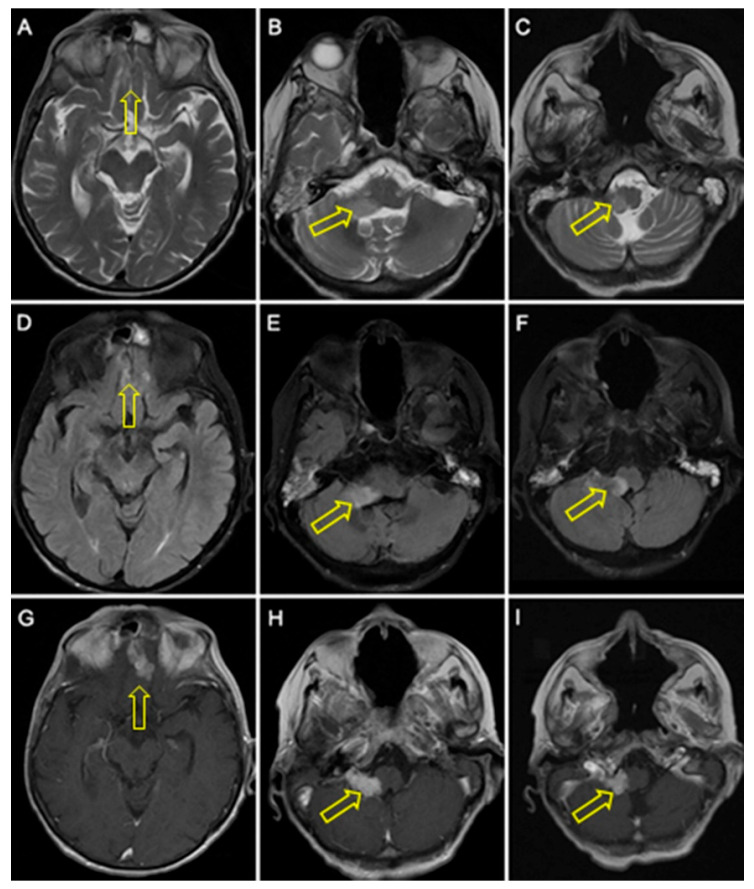
Granulomatosis with polyangiitis (GPA) is a rare necrotizing granulomatous vasculitis of small- to medium-sized vessels, typically associated with PR3-ANCA positivity, with an estimated prevalence of 20–160 cases/million [1,2]. While the upper respiratory tract, lungs, and kidneys are most commonly affected, nervous system involvement occurs in 22–54% of patients, predominantly affecting the peripheral nervous system. Central nervous system manifestations beyond cranial nerve palsies are uncommon and may arise from contiguous spread of granulomatous inflammation, small-vessel cerebral vasculitis, or primary intracranial granuloma formation [3]. Diagnosis relies on the combination of serology, imaging, and histopathology, with key differentials including other ANCA-associated vasculitides and infectious or malignant etiologies. Treatment follows a two-phase approach: induction with high-dose glucocorticoids, rituximab or cyclophosphamide, followed by maintenance immunosuppression to prevent relapse [4]. In October 2024, a 71-year-old female was admitted to the Emergency Center with episodic vomiting, severe headache, and gait instability for the last two months. Physical examination revealed a saddle-shaped nose, the presence of a hearing aid on the left side, a 15 mm oroantral communication, and two pinpoint perforations in the hard palate. These clinical findings are illustrated in Figure S1 in the Supplementary Materials. Her medical history revealed that non-specific respiratory symptoms had begun two years earlier, with radiographic findings initially interpreted as recurrent pneumonias. She was treated with multiple courses of broad-spectrum antibiotics, followed by antituberculosis therapy due to a borderline positive PCR for *Mycobacterium tuberculosis* and later antifungal therapy for suspected aspergillosis. During the disease course, she developed rapidly progressive sensorineural hearing loss. Subsequently, destructive changes in the hard palate occurred, requiring upper-jaw tooth extraction and resulting in a persistent oroantral communication. Computed tomography (CT) of the paranasal sinuses demonstrated extensive destruction of both nasal cavities, ethmoid sinuses, and the medial walls of the maxillary sinuses. Repeated ANCA testing was initially negative. Histopathological analysis of a polypoid nasal lesion showed necrosis with inflammatory infiltrate suggestive of a foreign-body granuloma; however, upon revision at our Clinic, the findings were consistent with necrotizing granulomatous inflammation with neutrophilic vasculitis and fibrinoid necrosis. Although histopathological images were not available, this case report primarily aims to highlight the radiological features of central nervous system involvement in GPA. Neurological examination upon admission revealed horizontal nystagmus during rightward gaze, bilateral hypoacusis, hypophonia, and dysphonia. Romberg’s test was positive, with the patient falling to the left with open eyes. Brain magnetic resonance imaging (MRI) revealed irregular meningeal thickening frontobasally bilaterally, more on the left (**A**,**D**,**G**—yellow arrow), and more pronounced in the region of the right middle cerebellar peduncle (**B**,**C**,**E**,**F**,**H**,**I**—yellow arrow), with the adjacent irregular-mass-like lesions in both regions. Lesions showed T2W (**A**–**C**) and FLAIR (**D**–**F**) hyperintensity with postcontrast enhancement (**G**–**I**). Considering the MRI features, the described lesions corresponded to granulomas. Additionally, destruction of the nasal septum, contents of the nasal cavities, ethmoid cells, and parts of the maxillary and sphenoid sinuses, with frontal sinusitis and chronic otomastoiditis on both sides, was noted (**B**,**C**). MRI findings in GPA patients with CNS involvement include cerebral and spinal cord pachymeningitis, cerebral ischemic lesions, cerebral hemorrhagic lesions, brain and spinal cord vessel abnormalities, pituitary gland enlargement, and granulomatous lesions of the brain and spinal cord. Most frequent clinical manifestations comprise severe headache, sensory and motor impairment, vestibular syndrome, hearing loss, and psychiatric disorders [5]. However, intracerebral granulomas are exceedingly rare and have been reported only sporadically [6,7]. The diagnosis was established in October 2024, with the patient meeting the classification criteria for GPA [8]. At this point, PR3-ANCA was only mildly elevated (38.4 IU/L, nv < 20 IU/L, ELISA), with no evidence of active lung or ENT disease, whereas CNS involvement was active and predominant. Eosinophil count was within the normal limits (0.1 × 10^9^/L, 0.6%), with no eosinophillia detected throughout the disease course to date. There were no signs of renal involvement. Previous small studies have shown that patients with GPA and severe CNS manifestations more frequently exhibit persistently negative ANCA antibodies compared to those with ‘classic’ GPA without CNS involvement [9].

**Figure 2:**
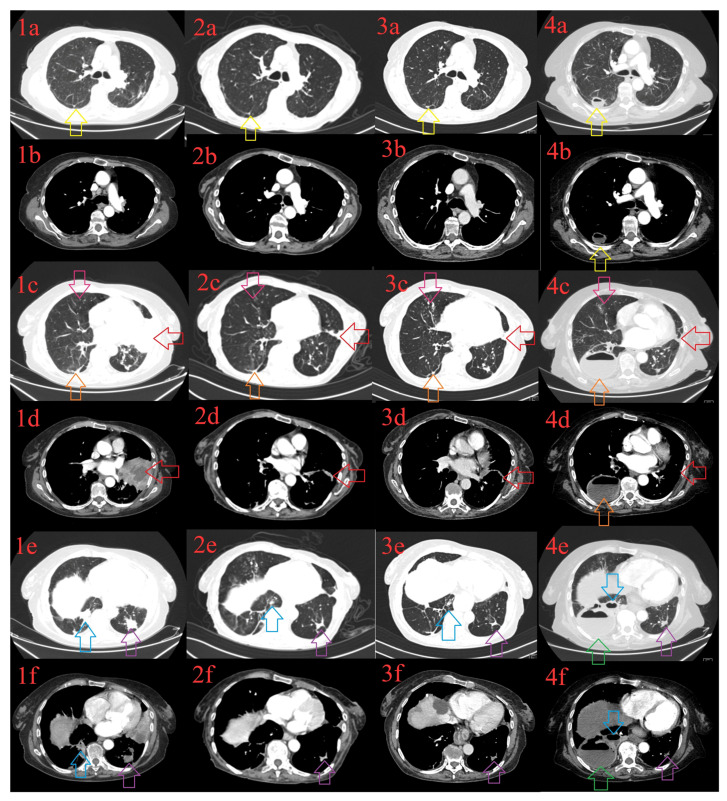
Figure 2 shows four consecutive chest CT examinations, with mutual time distances of 6, 3, and 6 months, throughout the disease course in the three sections of the chest in lung window (Images **1a**,**c**,**e**–**4a**,**c**,**e**) and soft tissue window (Images **1b**,**d**,**f**–**4b**,**d**,**f**), depicting the temporal versatility of caseous granulomatous lesions as a hallmark of GPA. At the level of bifurcation of trachea, in the posterior segment of the upper right lobe, there was a thin subpleural band with a solid nodule depicting mild size progression throughout three consecutive CT examinations (Images **1a**–**3a**, yellow arrow) that, after 6 months, became a caseous granuloma with excavation (**4a**,**4b**, yellow arrow). At the level of the left atrium, in the lingula of the upper left lobe, there was caseous consolidation (**1c**,**1d**, red arrow), previously misunderstood as an excavating carcinoma, that became a thick subpleural band in the following three CT examinations (**2d**–**4d**, red arrow). At the same level, in the apicobasal segment of the lower right lobe, there was a thick subpleural band with a solid nodule in three consecutive CT examinations (**1c**–**3c**, orange arrow) that, after 6 months, became a caseous granuloma with excavation (**4c**,**4d**, orange arrow). Also, there was development of centrilobular nodules in a “tree-in-bud” pattern in the middle lobe throughout four consecutive chest CT examinations (**1c**–**4c**, pink arrow). At the level of the left ventricle, in the lower right lobe in medio-basal segment, there was caseous granuloma in resolution (**1e**,**1f**, blue arrow) that became a thick subpleural band in the two following CT examinations (**2e**,**3e**, blue arrow) but evolved to a larger caseous granuloma after six months (**4e**,**4f**, blue arrow), with development of similar lesions in the neighboring posterobasal segment (**4e**,**4f**, green arrow). At the same level, in the laterobasal segment of the lower left lobe, there was a caseous granuloma (**1e**,**1f**, purple arrow), which became a thick subpleural band in the following three CT examinations (Images **2e**,**f**–**4e**,**f**, purple arrow). Additional lung CT images are provided in Figures S2 and S3 (Supplementary Materials).

**Figure 3:**
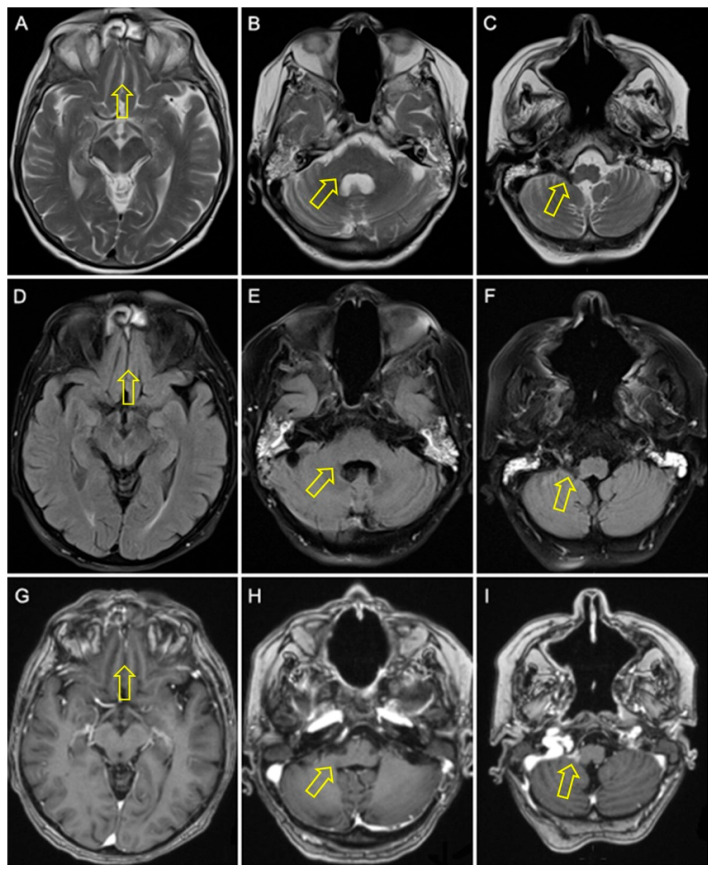
The patient received pulse methylprednisolone therapy (total 1.25 g) and a single pulse dose (0.5 g/m^2^) of cyclophosphamide (CYC). The treatment was continued with rituximab (RTX) at a dose of 375 mg/m^2^ administered in four weekly doses, leading to significant clinical improvement. A follow-up brain MRI performed in January 2025 revealed significant regression, with small residual lesions frontobasally bilaterally and in the right cerebellomedullary cistern. MRI revealed discrete irregular frontobasal meningeal thickening (**A**,**D**,**G**—yellow arrow), and small irregular lesion in the region of the right middle cerebellar peduncle (**B**,**C**,**E**,**F**,**H**,**I**—yellow arrow), without T2W (**A**–**C**) and FLAIR (**D**–**F**) hyperintensity, but with discrete postcontrast enhancement (**G**–**I**). Standard induction with corticosteroids and CYC is effective in most GPA patients with CNS involvement [5]. However, in refractory or relapsing cases, RTX has shown superior efficacy [5]. Therapy was continued with low doses of prednisone and methotrexate (0.3 mg/kg BW weekly), with no new neurological symptomatology observed during the follow-up period. In July 2025, the patient experienced worsening of respiratory symptoms, including a persistent cough and new-onset hemoptysis. Lung CT showed active lung involvement with formation of caseous granulomas (Figure 2, Images 4a–4f). The patient received two pulse doses of intravenous methylprednisolone (500 mg each), followed by RTX at a dose of 1000 mg administered twice, two weeks apart. The treatment resulted in a good clinical response, with sustained disease remission to date. This case highlights the diagnostic challenge posed by intracerebral granulomas as a rare manifestation of GPA in a patient with unremarkable ANCA serology. The tumor-like appearance of intracranial lesions on MRI may lead to significant diagnostic delay. Multimodal imaging, integrating brain MRI with sinonasal and pulmonary CT findings, proved essential in establishing the correct diagnosis. This case reinforces the importance of including GPA in the differential diagnosis of intracranial mass lesions and underscores that early initiation of immunosuppressive therapy is critical to prevent life-threatening compressive complications.

## Data Availability

The original contributions presented in this study are included in the article/Supplementary Material. Further inquiries can be directed to the corresponding author.

## Supplementary Materials

The following supporting information can be downloaded at https://www.mdpi.com/article/10.3390/diagnostics16142277/s1, Figure S1: Clinical photograph demonstrating a saddle-shaped nose deformity, a 15-mm oroantral communication, and two pinpoint perforations in the hard palate; Figure S2: Four consecutive chest CT examinations, with mutual time distances of 6, 3, and 6 months, in curved planar reformation of the chest in lung window (Images 1–4a) and soft tissue window (Images 1–4b), depict the temporal versatility of caseous granulomatous lesions. At the end of bronchus for the mediobasal segment of the lower right lobe, there is caseous granuloma (Images 1a and 1b, arrow), that becomes thick pleural band, in the following two CT, (Images 2–3a and b, arrow), in order to exacerbate in larger caseous lesion, after six months (Images 4a and 4b, arrow). Also, at the level of the origin of the bronchus for lingula of the upper left lobe, there is caseous consolidation (Images 1a and 1b, arrow), which does not resolve in entirety, in the following CT (Images 2–4a and b, arrow), while its periphery becomes a thick subpleural band (Images 2–4a, arrow); Figure S3: Four consecutive chest CT examinations, with mutual time distance of 6, 3 and 6 months, in curved planar reformation (Images 1–4a) and trans-axial reformation (Images 1–4b). In the middle lobe, there is development of irregular centrilobular nodules distributed in “tree-in-bud” pattern, surrounded by ground-glass opacities and septal interstitial thickening (Images 1–4a and b, red arrow), with calcified degeneration in the following three CT (Images 2–4a and b, green arrow).
