# Supplementary material for: Uncommon Territory: Multifocal Tumor-like Brain Lesions in Granulomatosis with Polyangiitis
Source: Diagnostics (Basel). 2026 Jul 21;16(14):2277. doi: 10.3390/diagnostics16142277 (PMC13408036; doi:10.3390/diagnostics16142277)
Supplement: Supplementary file 1 [file diagnostics-16-02277-s001.zip › diagnostics-4447768-supplementary.pdf]

Supplementary Materials

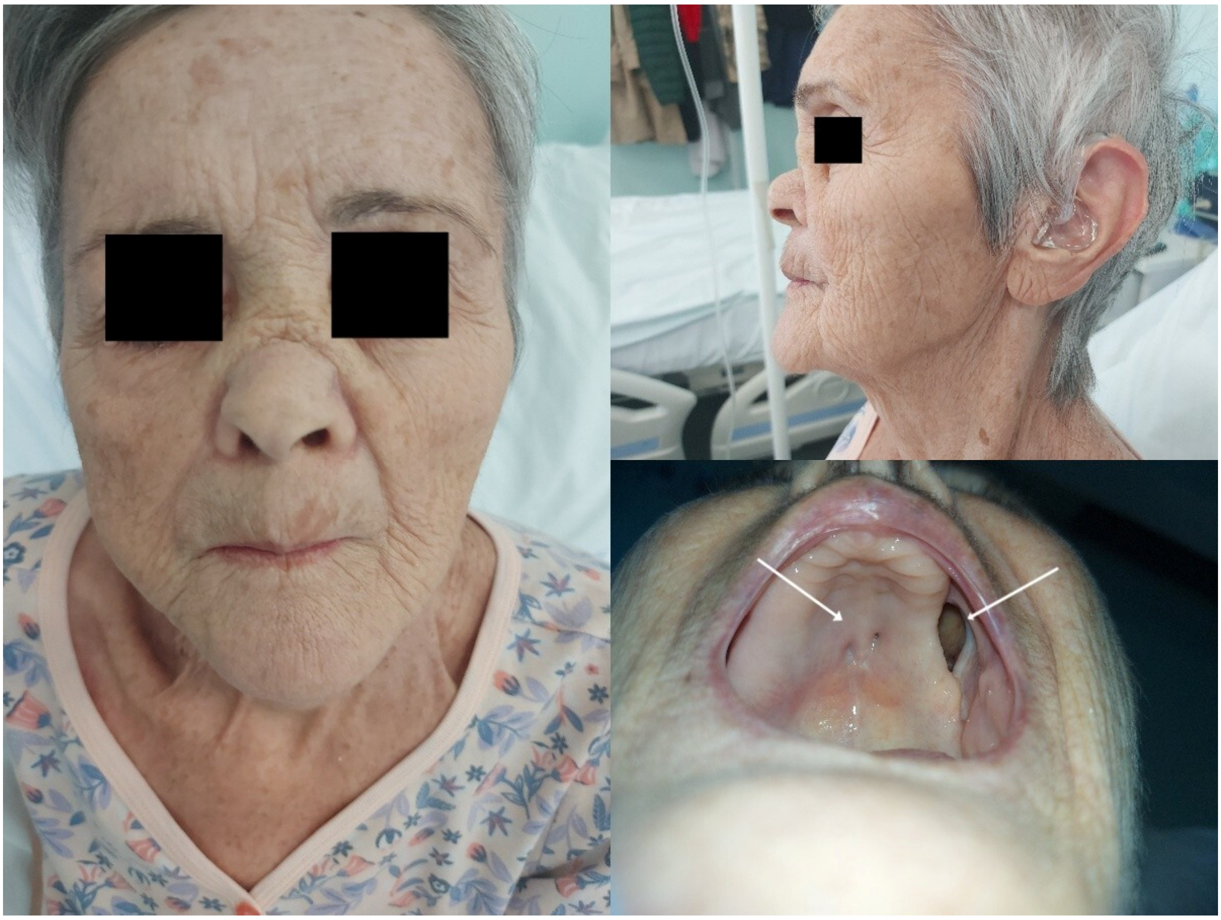

Figure S1. Clinical photograph demonstrating a saddle-shaped nose deformity, a 15-mm oroantral communication, and two pinpoint perforations in the hard palate

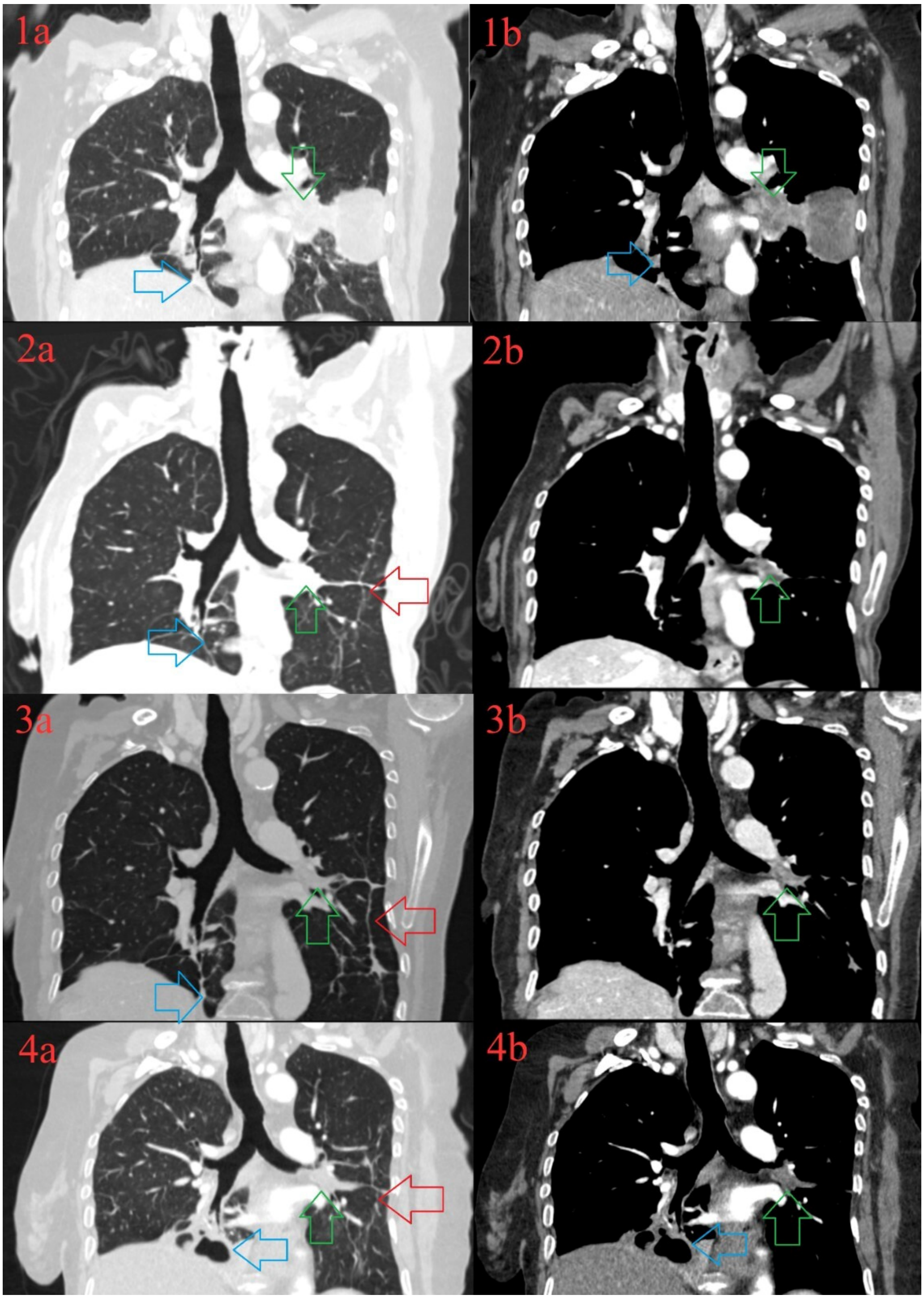

Figure S2. Four consecutive chest CT examinations, with mutual time distances of 6, 3, and 6 months, in curved planar reformation of the chest in lung window (Images 1-4a) and soft tissue window (Images 1-4b), depict the temporal versatility of caseous granulomatous lesions. At the end of bronchus for the mediobasal segment of the lower right lobe, there is caseous granuloma

(Images 1a and 1b, arrow), that becomes thick pleural band, in the following two CT, (Images 2-3a and b, arrow), in order to exacerbate in larger caseous lesion, after six months (Images 4a and 4b, arrow). Also, at the level of the origin of the bronchus for lingula of the upper left lobe, there is caseous consolidation (Images 1a and 1b, arrow), which does not resolve in entirety, in the following CT (Images 2-4a and b, arrow), while its periphery becomes a thick subpleural band (Images 2-4a, arrow).

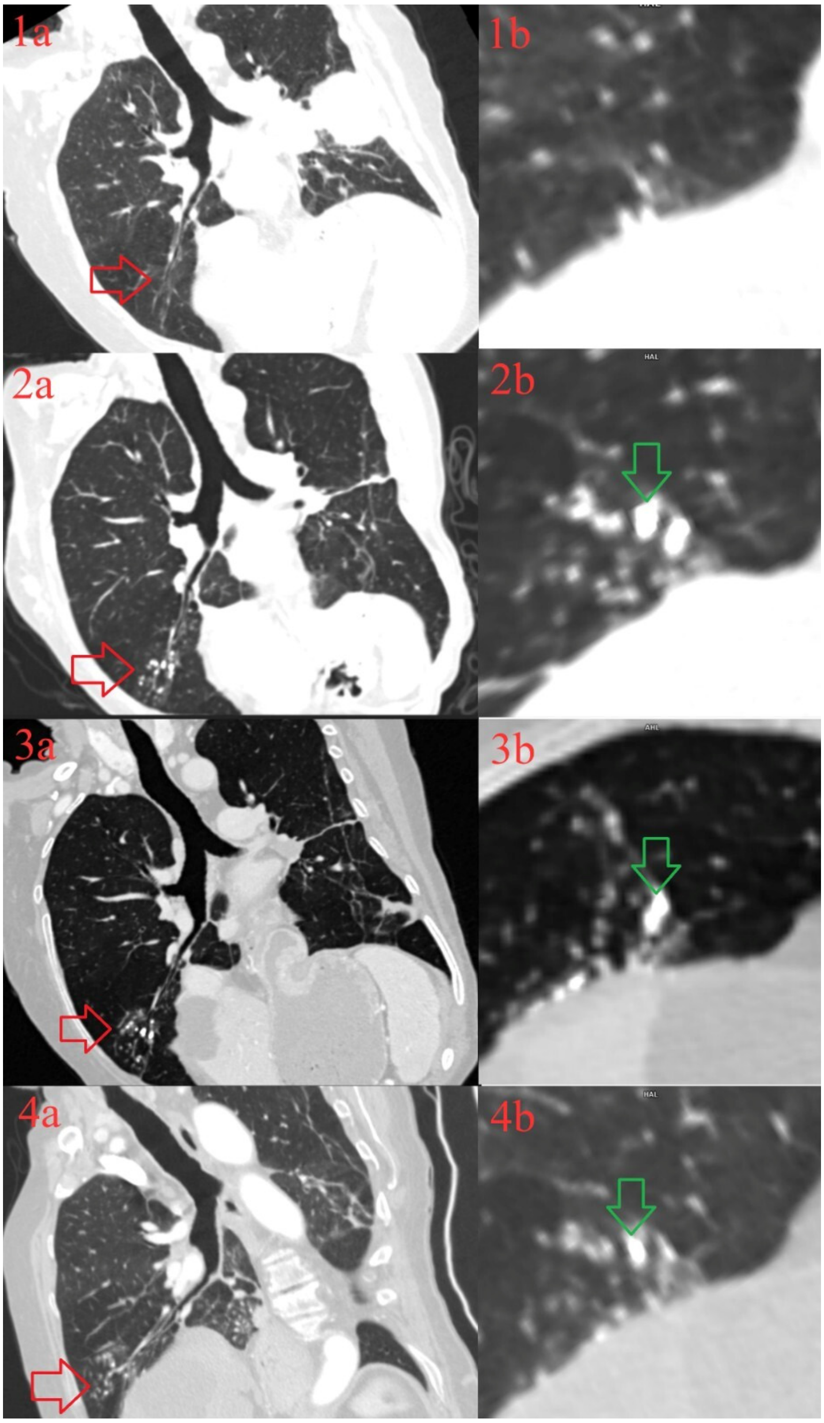

Figure S3. Four consecutive chest CT examinations, with mutual time distance of 6, 3 and 6 months, in curved planar reformation (Images 1-4a) and trans-axial reformation (Images 1-4b). In the middle lobe, there is development of irregular centrilobular nodules distributed in “tree-in-bud” pattern, surrounded by ground-glass opacities and septal interstitial thickening (Images 1-4a and b, red arrow), with calcified degeneration in the following three CT (Images 2-4a and b, green arrow).
